# Supplementary material for: A unified Watson-Crick geometry drives transcription of six-letter expanded DNA alphabets by E. coli RNA polymerase
Source: Nat Commun. 2023 Dec 12;14:8219. doi: 10.1038/s41467-023-43735-9 (PMC10716388; doi:10.1038/s41467-023-43735-9)
Supplement: Supplementary file 1 — Supplementary Information [file 41467_2023_43735_MOESM1_ESM.pdf]

Supplementary Information for

A Unified Watson-Crick Geometry Drives Transcription of Six-Letter Expanded DNA Alphabets by *E. coli* RNA Polymerase

Juntaek Oh[1,8,9], Zelin Shan[2,9], Shuichi Hoshika[3,9], Jun Xu[1], Jenny Chong[1], Steven A. Benner[3,*], Dmitry Lyumkis[2,4,5*,] Dong Wang[1,6,7*]

[1]Division of Pharmaceutical Sciences, Skaggs School of Pharmacy & Pharmaceutical Sciences; University of California, San Diego, La Jolla, California 92093, United States

[2] The Salk Institute for Biological Studies, La Jolla, CA 92037, United States.

[3] Foundation for Applied Molecular Evolution, 13709 Progress Blvd Box 7, Alachua, FL 32615, United States

[4] Department of Integrative Structural and Computational Biology, The Scripps Research Institute 10550 N Torrey Pines Rd, La Jolla, CA 92037, United States.

[5] Graduate School of Biological Sciences, Section of Molecular Biology, University of California San Diego, La Jolla, CA 92093, United States.

[6] Department of Cellular and Molecular Medicine, University of California, San Diego, La Jolla, California 92093, United States

[7] Department of Chemistry and Biochemistry, University of California, San Diego, La Jolla, California 92093, United States

[8] Department of Pharmacy, College of Pharmacy, Kyung Hee University; Seoul 02447, Republic of Korea

[9] These authors are equally contributed.

[*] To whom correspondence should be addressed. Email: SAB: sbenner@ffame.org; DL: dlyumkis@salk.edu; DW: dongwang@ucsd.edu

Supplementary Figures

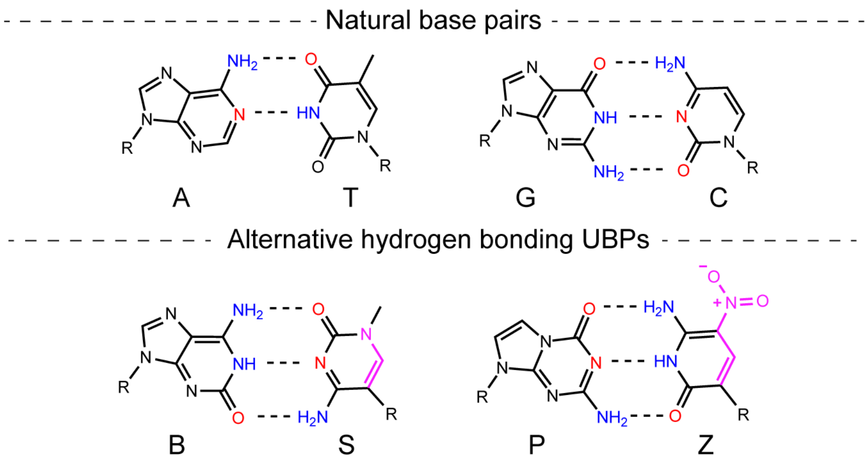

Supplementary Figure 1. Schematic representation of natural base pair and AEGIS base pair (B, S, P, Z). Natural hydrogen bonding pairs and unnatural, alternative hydrogen bonding pairs (**B:S, P:Z**) are shown. **P:Z** pair refers the base pair between 2-amino-imidazo-[1,2a]-1,3,5-triazin-[8H]-4-one with 6-amino-3-5-nitro-1H-pyridin-2-one.

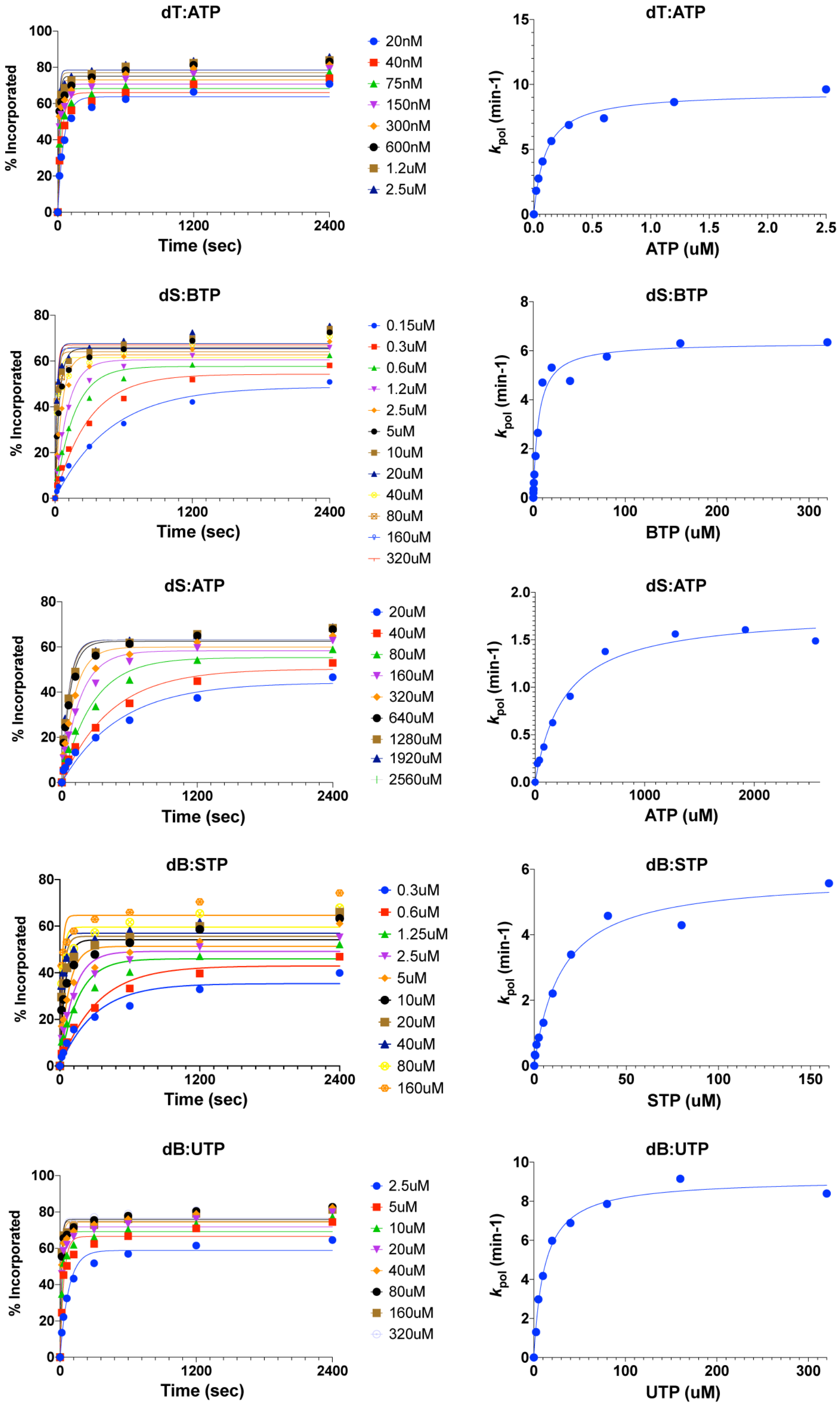

Supplementary Figure 2. Single turn-over nucleotide incorporation kinetic analysis of natural and unnatural base pair. Kinetic parameters were calculated from biochemical assays with incubation time ranging from 0 to 40 min (0 sec, 15 sec, 30 sec, 1 min, 2 min, 5 min, 10

min, 20 min, 40 min). The substrates and their concentrations for each assay are indicated in each corresponding plot. Regression curves were generated using Prism 8.

dT_ATP dT_ATP

Time 0 15''30''1' 2' 5' 10' 20' 40'

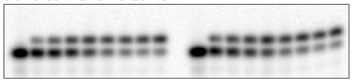

20nM 40nM

dT_ATP dT_ATP dT_ATP

Time 0 15''30''1' 2' 5' 10' 20' 40'

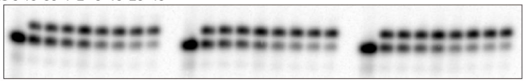

75nM 150nM 300nM

dT_ATP dT_ATP dT_ATP

Time 0 15''30''1' 2' 5' 10' 20' 40'

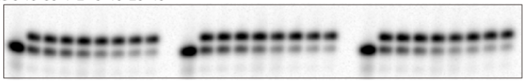

600nM 1200nM 2500nM

dS_BTP dS_BTP

Time 0 15''30''1' 2' 5' 10' 20' 40'

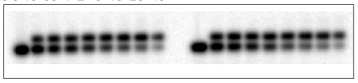

40uM 80uM

dS_BTP dS_BTP dB_STP

Time 0 15''30''1' 2' 5' 10' 20' 40'

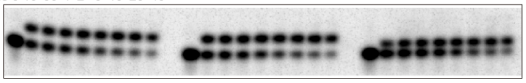

160uM 320uM 80uM

dB_STP dB_STP

Time 0 15''30''1'2'5'10 20'40'

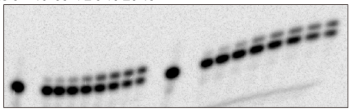

600nM 300nM

dB_STP dB_STP dB_STP

Time 0 15''30''1' 2' 5' 10' 20' 40'

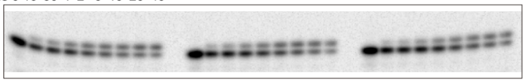

5uM 2.5uM 1.25uM

dB_STP dB_STP dB_STP

Time 0 15''30''1' 2' 5' 10' 20' 40'

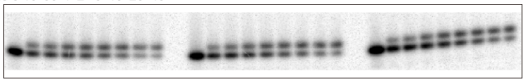

40uM 20uM 10uM

dS_BTP dS_BTP dS_BTP

Time 0 15''30''1' 2' 5' 10' 20' 40'

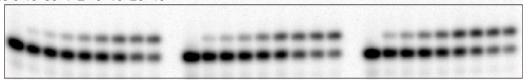

150nM 300nM 600nM

dS_BTP dS_BTP dS_BTP

Time 0 15''30''1' 2' 5' 10' 20' 40'

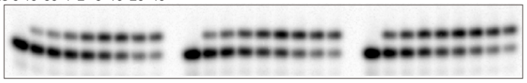

1.2uM 2.5uM 5uM

dS_BTP dS_BTP dS_ATP

Time 0 15''30''1' 2' 5' 10' 20' 40'

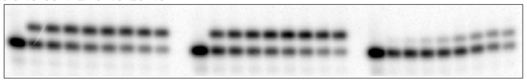

10uM 20uM 20uM

dS_ATP dS_ATP dS_ATP

Time 0 15''30''1' 2' 5' 10' 20' 40'

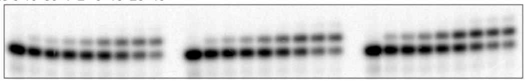

40uM 80uM 160uM

dS_ATP dS_ATP

Time 0 15''30''1' 2' 5' 10' 20' 40'

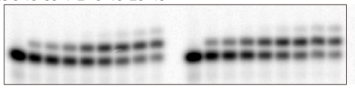

320uM 640uM

dS_ATP dS_ATP dS_ATP

Time 0 15''30''1' 2' 5' 10' 20' 40'

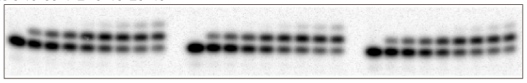

1280uM 1960uM 2560uM

dB_STP dB_UTP dB_UTP

Time 0 15''30''1' 2' 5' 10' 20' 40'

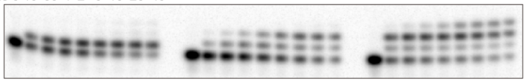

160uM 2.5uM 320uM

dB_UTP dB_UTP dB_UTP

Time 0 15''30''1' 2' 5' 10' 20' 40'

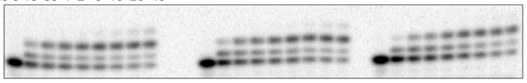

20uM 10uM 5uM

dB_UTP dB_UTP dB_UTP

Time 0 15''30''1' 2' 5' 10' 20' 40'

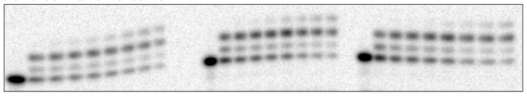

160uM 80uM 40uM

Supplementary Figure 3. Raw gel data for single turn-over nucleotide incorporation assay. Raw gel data for Supplementary Figure 2. All samples were denatured by heating at 95°C for 10 minutes and subsequently subjected to analysis using a 12% denaturing urea-PAGE gel.

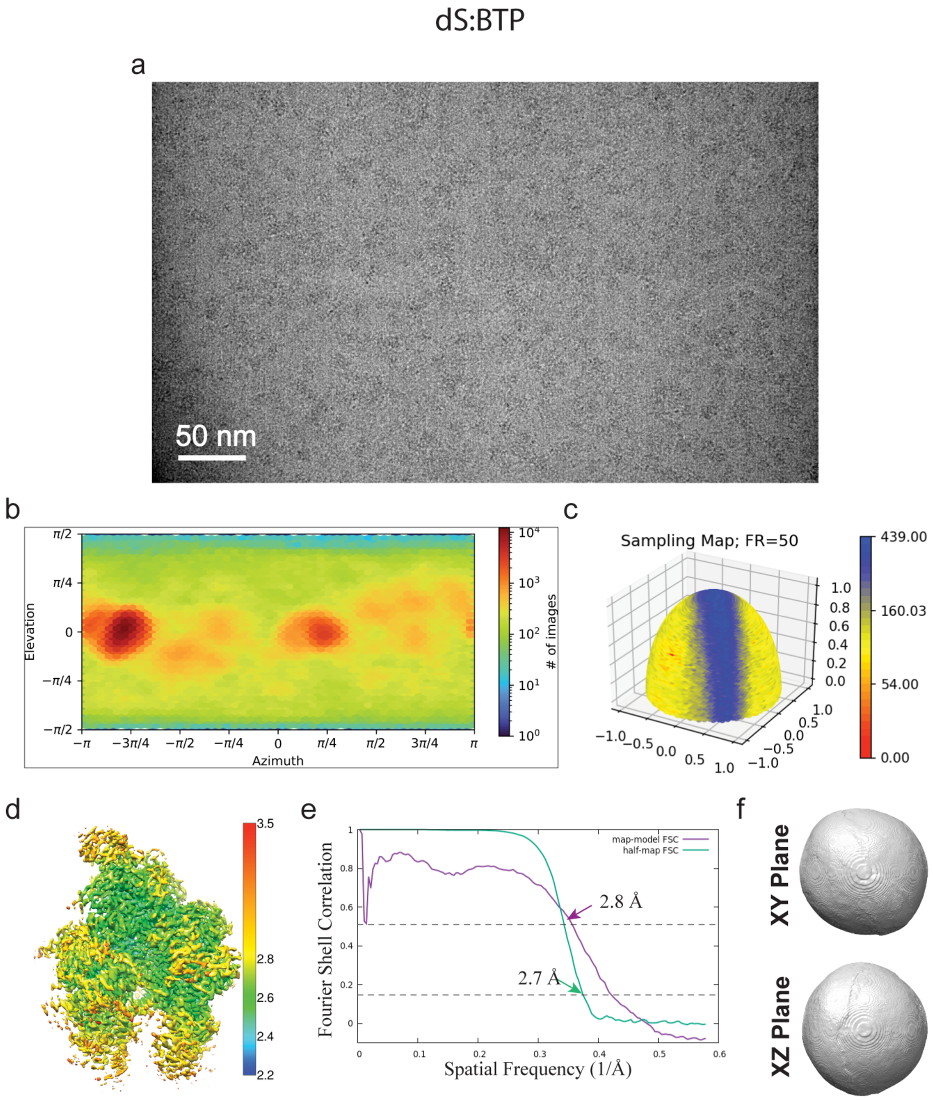

Supplementary Figure 4. Cryo-EM validation of the RNAP bound to dS:BTP reconstructions. (a) A Cryo-EM micrograph represents the particle distribution on an UltrAufoil gird. (b) Euler angle plot showing the distribution of projection orientations used in the cryo-EM reconstruction. (c) Surface sampling plot of the Fourier voxel sampling derived from the Euler angle distribution plot, calculated with a Fourier radius set to 50 voxels. The sampling compensation factor (SCF) is indicated in Table S1 (1, 2). (d) Cryo-EM reconstruction colored by local resolution. (e) Fourier shell correlation (FSC) curves derived from half-map and map-to-model reconstructions, with FSC cutoffs 0.143 and 0.5 indicated, respectively, as well as nominal resolution values. (f) 3DFSC (3) shown as an isosurface and thresholded using a value of 0.5 with two perpendicular planar views.

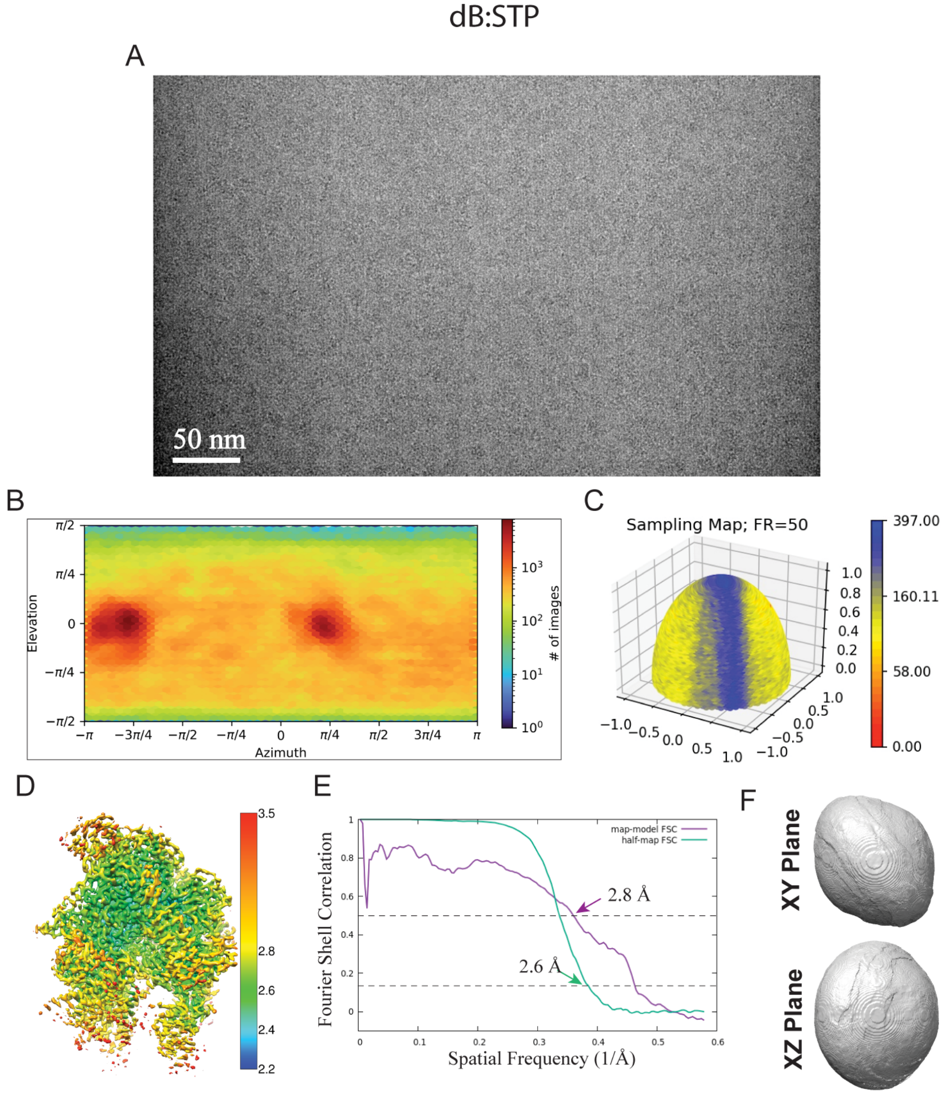

Supplementary Figure 5. Cryo-EM validation of the RNAP bound to dB:STP reconstructions. (a) A Cryo-EM micrograph represents the particle distribution on an UltrAufoil gird. (b) Euler angle plot showing the distribution of projection orientations used in the cryo-EM reconstruction. (c) Surface sampling plot of the Fourier voxel sampling derived from the Euler angle distribution plot, calculated with a Fourier radius set to 50 voxels. The sampling compensation factor (SCF) is indicated in Table S1 (1, 2). (d) Cryo-EM reconstruction colored by local resolution. (e) Fourier shell correlation (FSC) curves derived from half-map and map-to-model reconstructions, with FSC cutoffs 0.143 and 0.5 indicated, respectively, as well as nominal resolution values. (f) 3DFSC (3) shown as an isosurface and thresholded using a value of 0.5 with two perpendicular planar views.

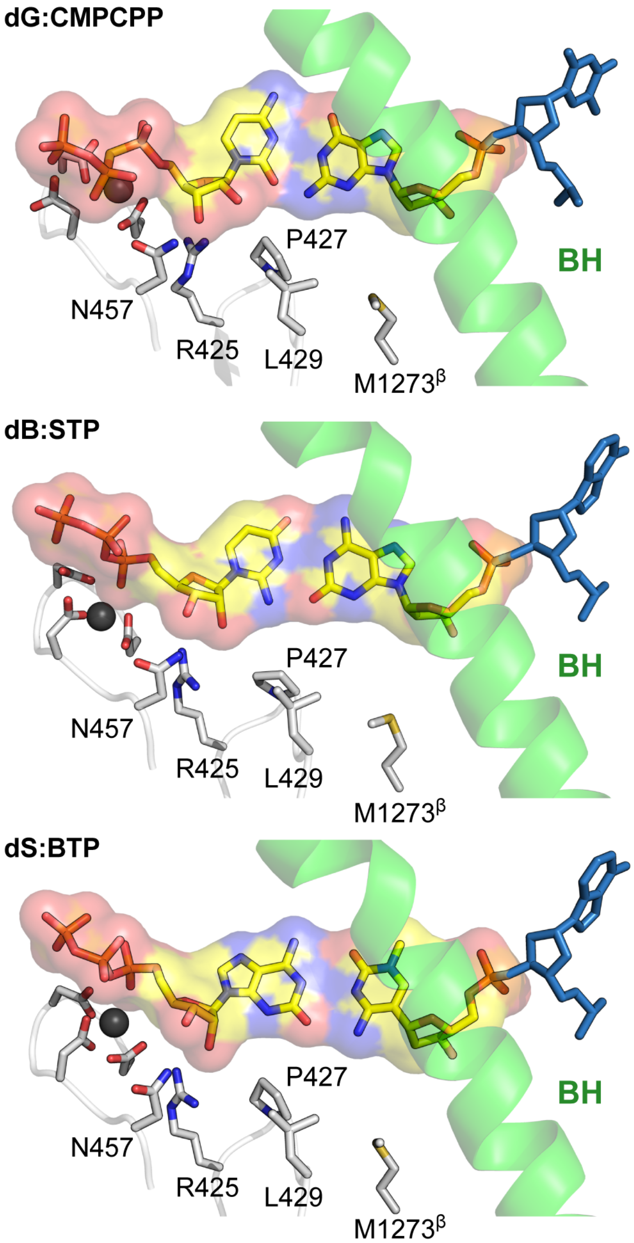

Supplementary Figure 6. Minor groove interaction between *E. coli* RNAP and dG:CMPCPP / dB:STP / dS:BTP. UBP or natural base pair are shown together with key minor groove residues of RNAP. Bridge helix is colored in green. Bases in +1 position are colored in yellow. +2 template base is colored in blue. Other interacting residues are colored in white. Note that M1273 is from β-subunit and R425, P427, L429 and N457 is from β'-subunit. PDB code for dG:CMPCPP is 7MKO[https://doi.org/10.2210/pdb7MKO/pdb](E.coli RNA polymerase elongation complex). The color code is the same as Figure 5.

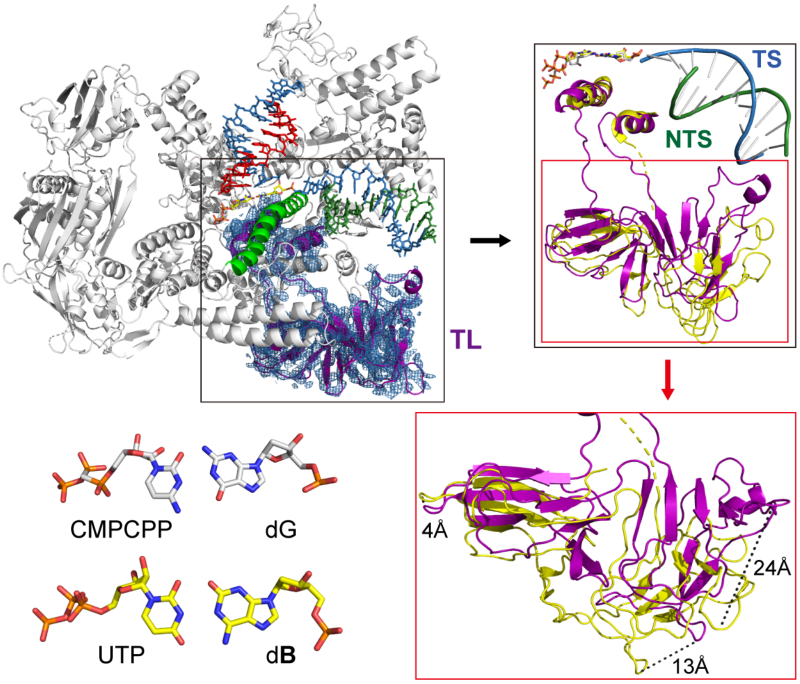

Supplementary Figure 7. *E. coli* RNAP trigger loop insertion domain comparison. Trigger loop of dB:UTP and dG:CMPCPP structures are superposed for comparison. While the two helix bundles near the bridge helix superimposed well, the insertion domain (yellow (dG:CMPCPP structure) and purple (dB:UTP structure)) show 4~24 Å shift from each other. Other color code is the same as Figure 2.

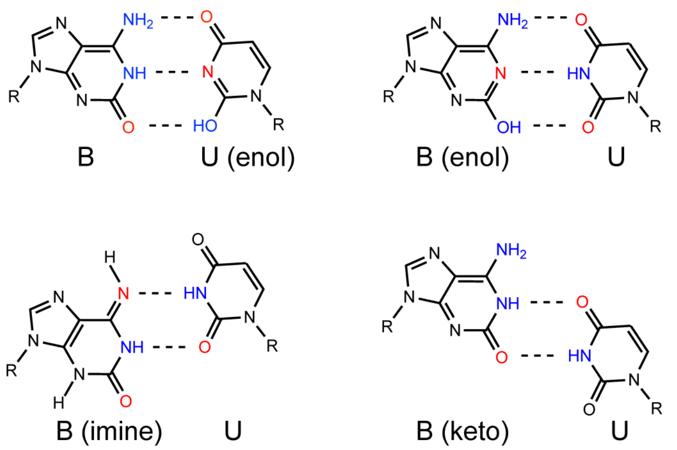

Supplementary Figure 8. Schematic representation of potential B:U mismatched pair. Base paring scheme of B:U (enol tautomer), B (enol tautomer):U, B (imine tautomer):U wobble base pair and B (keto):U reverse wobble base pairs are shown.

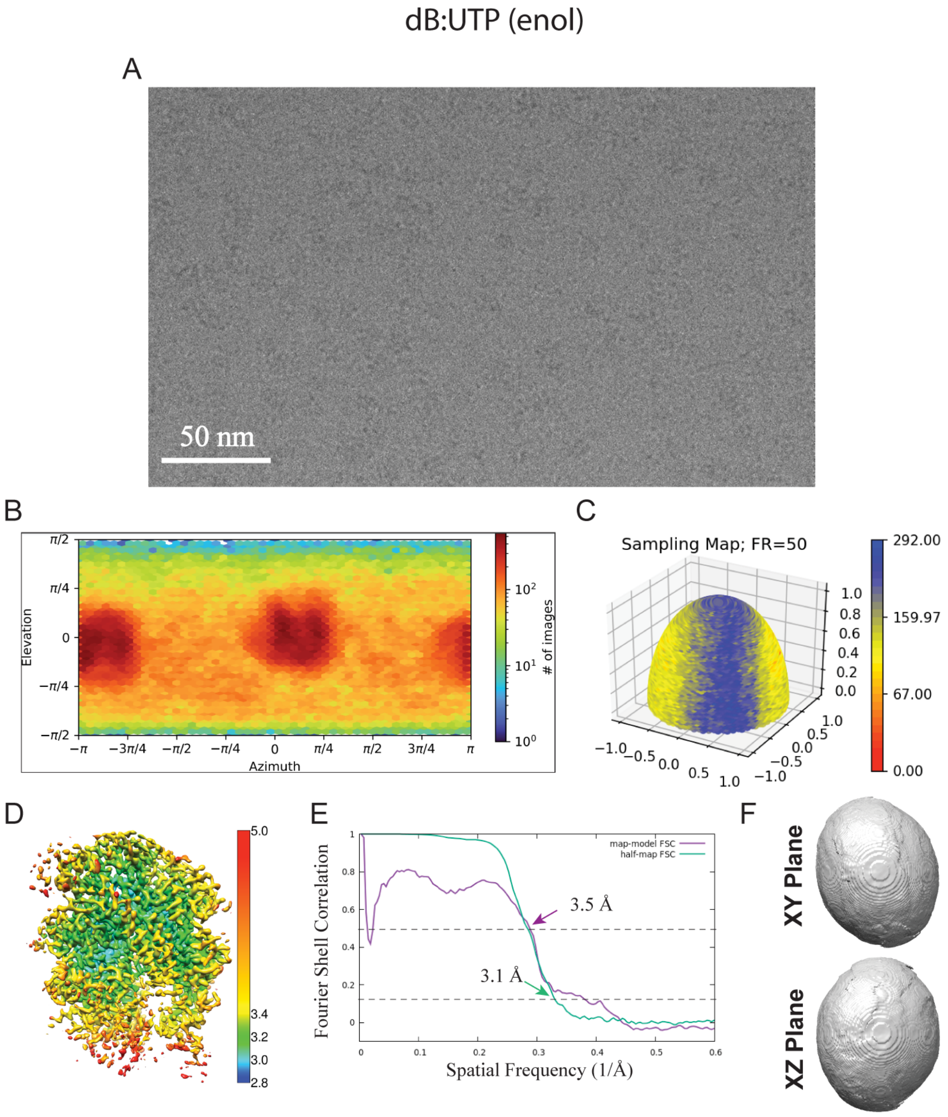

Supplementary Figure 9. Cryo-EM validation of the RNAP bound to dB:UTP (enol) reconstructions. (a) A Cryo-EM micrograph represents the particle distribution on an UltrAufoil gird. (b) Euler angle plot showing the distribution of projection orientations used in the cryo-EM reconstruction. (c) Surface sampling plot of the Fourier voxel sampling derived from the Euler angle distribution plot, calculated with a Fourier radius set to 50 voxels. The sampling compensation factor (SCF) is indicated in Supplementary Table 1 (1, 2). (d) Cryo-EM reconstruction colored by local resolution. (e) Fourier shell correlation (FSC) curves derived from half-map and map-to-model reconstructions, with FSC cutoffs 0.143 and 0.5 indicated, respectively, as well as nominal resolution values. (f) 3DFSC (3) shown as an isosurface and thresholded using a value of 0.5 with two perpendicular planar views.

Supplementary Table 1. Cryo-EM Data and Structure Refinement Statistics

Data collection and processing	dS:BTP	dB:STP	dB:UTP(enol)
Microscope	Titan Krios G3i(SLAC)	Titan Krios G3i(SLAC)	Titan Krios G2
Voltage (keV)	300	300	300
Camera	Gatan K3	Gatan K3	Gatan K2 Summit
Magnification	105K	105K	165K
Pixel size at detector(Å/pixel)	0.86	0.86	0.83
Total fluence (e–/Å2)	30.0	35.0	33.8
fluence rate (e–/pixel/sec)	23.6	17.1	7.3
Exposure time (sec)	0.9	1.5	3.2
Number of frames	60	70	40
Defocus range (µm)	0.8-2.6	1.0-2.4	1.0-2.5
Automation software	EPU	EPU	Leginon
Energy filter slit width	20	20	
Stage tilt (°)	0 and 20	0 and 20	0
No. of movies	8,598	11,268	5,300
Total extracted particles	2,087,449	3,435,744	544,816
No. of particles in final map	1,032,305	1,326,857	268,973
Resolution, Fourier shell correlation 0.143 (Å)	2.70	2.65	3.28
Sampling Compensation Factor (SCF)	0.79	0.87	0.91
Directional resolution range from 3D FSC (Å)	2.6-2.8	2.5-2.8	3.1-3.6

(continue)

**Atomic model refinement**	**dS:BTP**	**dB:STP**	**dB:UTP (enol)**
**Initial model**	6ALH	dS:BTP	dS:BTP
**Composition**			
Non-hydrogen atoms	21415	21160	23375
Protein residues	2682	2737	2922
Nucleotide residues	43	41	41
Waters	375	413	0
Ligands	5	5	5
**B-factors** (Å^2)			
Protein	72.5	93.0	119.2
Nucleotide	120.2	203.5	183.5
Ligand	72.7	119.8	154.5
**R.m.s. deviations**			
Bond lengths (Å)	0.003	0.003	0.002
Bond angles (°)	0.542	0.615	0.548
**Validation**			
MolProbity score	1.30	1.48	1.56
Clashscore	2.91	4.92	7.49
Rotamer outliers (%)	0	0	0
Ramachandran outliers (%)	0.08	0.04	0
**PDB ID**	8SY5	8SY7	8SY6
**EMDB ID**	EMD-40862	EMD-40864	EMD-40863

Supplementary References

1. P. R. Baldwin, D. Lyumkis, Non-uniformity of projection distributions attenuates resolution in Cryo-EM. *Prog Biophys Mol Biol* **150**, 160-183 (2020).
2. P. R. Baldwin, D. Lyumkis, Tools for visualizing and analyzing Fourier space sampling in Cryo-EM. *Prog Biophys Mol Biol* **160**, 53-65 (2021).
3. Y. Z. Tan *et al.*, Addressing preferred specimen orientation in single-particle cryo-EM through tilting. *Nat Methods* **14**, 793-796 (2017).
